# The Potential of Artificial Intelligence, Machine Learning, and Novel Analytic Methods to Promote Successful Aging

**DOI:** 10.1093/geroni/igaa057.2259

**Published:** 2020-12-16

**Authors:** Walter Boot

## Abstract

The Gerontological Society of America is celebrating its75th anniversary and in those75 years the world has undergone an amazing technological revolution. During this period, computers transformed from systems that once filled entire rooms to much more powerful devices that fit in our pockets. We have seen the introduction of wireless technologies, augmented and virtual reality, smart home devices, autonomous vehicles, and much more. This session focuses on a new technological advance that has the potential to support the health, wellbeing, and independence of older adults and caregivers: artificial intelligence (AI). This session will present applications of AI, Machine Learning (ML), and other novel analytic methods and how they have the potential to impact the lives of older adults in a variety of context. As AI is increasingly being involved in workplace hiring, the first talk focuses on older adults’ attitudes toward the role of AI in this decision making process. Next, novel ML approaches applied to social media are discussed in terms of understanding the needs of Alzheimer’s caregivers. Next, ML techniques are discussed in terms of developing biomarkers that can be applied in diagnosis and assessment of therapeutic responses by detecting mood, which may have important implications for older adults living with dementia. Then, the potential role of AI is discussed in terms of developing reminder systems to promote older adults’ adherence to technology-based health activities. Finally, novel analytic approaches are discussed in terms of harnessing digital metrics to detect the risk of cognitive decline.

